# Supplementary material for: MTSv: rapid alignment-based taxonomic classification and high-confidence metagenomic analysis
Source: PeerJ. 2022 Nov 8;10:e14292. doi: 10.7717/peerj.14292 (PMC9651046; doi:10.7717/peerj.14292)
Supplement: Supplemental Information 2 [file peerj-10-14292-s002.docx]

Supplemental Methods

# *Francisella tularensis* Ft-sp.FTS_0772 assay

This TaqMan real-time PCR assay targets a *F. tularensis-*specific signature region from 1187646–1187807 of the reference SchuS4 whole genome sequence (AJ749949.2) (Öhrman et al, 2021). The assay was run with a 10μl volume and contained: 1 x TaqMan Universal PCR master mix (Life Technolgies Appplied Biosystems, Foster City) 0.9μM Primers (Integrated DNA Technolgies, San Diego, CA, USA), 0.2μM Probe (Life Technologies, Applied Biosystems, Foster City, CA) and 1μl of DNA (1ng). The thermal cycle conditions were: 50ºC for 2 min, 95ºC for 10 min, and 50 cycles of 95ºC for 15 sec and 60ºC for 1 min. Assays were run on a Life Technologies Quant Studio Instruments Flex Real-Time PCR System.

Forward Primer: 5’ – CAAGGTAAAGAAATTAAGAGTAGTAAAGTTGAATTC – 3’

Reverse Primer: 5’ – ATTTAATCTAGTATTATCAATTGGGTAAAAAGGTA – 3’

Probe: FAM-GATGTGGCAACAACTGAAAT (6-carboxyfluorescein (FAM) fluorophore and a 3’ black hole quencher-1)

A minor groove binding moiety was added to the probe to increase the melting temperature and stabilize hybridization.

# *Bacillus anthracis* assay

The assay targeted a nonsense mutation in the *B. anthracis plcR* gene. The assay was a TaqMan-minor groove binding allelic discrimination assay around the nonsense mutation (Easterday et al., 2005). The assay was run with a 10μl reaction (see above). The thermal cycle conditions were 50ºC for 2 min 95ºC for 2 min, and 40 cycles of 95ºC for 15 sec and 60ºC for 1 min.

Forward Primer: 5’ – CCAATCAATGTCATACTATTAATTTGACAC – 3’

Reverse Primer: 5’ – ATGCAAAAGCATTATACTTGGACAAT – 3’

Probe: 5′-VIC-CAAAGCGCTTATTCGTATT-3′-MGB

# *Yersinia pestis* assay

The assay targeted the plasminogen activator (*pla*) gene. See above *B. anthracis* assay for reaction mixtures and thermal cycle conditions.

Forward Primer: 5’ – ATCTTACTTTCCGTGAGAAG – 3’

Reverse Primer: 5’ – CTTGGATGTTGAGCTTCCTA – 3’

Probe: 5’ – ATACTGTGACGGCGGGTCTG – 3’

Easterday WR, Van Ert MN, Simonson TS, Wagner DM, Kenefic LJ, Allender CJ, Keim P. 2005. Use of single nucleotide polymorphisms in the *plcR* gene for specific identification of *Bacillus anthracis*. *Journal of Clinical Microbiology* 43:1995–1997. DOI: 10.1128/JCM.43.4.1995-1997.2005.

Hinnebusch J, Schwan TG. 1993. New method for plague surveillance using polymerase chain reaction to detect *Yersinia pestis* in fleas. *Journal of Clinical Microbiology* 31:1511–1514. DOI: 10.1128/jcm.31.6.1511-1514.1993.

Liu CM, Aziz M, Kachur S, Hsueh PR, Huang YT, Keim P, Price LB. 2012. BactQuant: an enhanced broad-coverage bacterial quantitative real-time PCR assay. *BMC Microbiology* 12:56. DOI: 10.1186/1471-2180-12-56.
